# Diagnostic Challenges of Early Mycosis Fungoides in Skin of Color: A Case Report and Review

**DOI:** 10.7759/cureus.83081

**Published:** 2025-04-27

**Authors:** Paige O Daly, Patricia Wessler

**Affiliations:** 1 Research, Edward Via College of Osteopathic Medicine, Blacksburg, USA; 2 Family Medicine, Riverside Regional Medical Center, Newport News, USA

**Keywords:** case report, cutaneous t-cell lymphoma (ctcl), dermatology, diagnostic delay, early-stage mycosis fungoides, family medicine, immunohistochemistry, mycosis fungoides (mf), skin biopsy

## Abstract

Mycosis fungoides (MF), the most common type of primary cutaneous T-cell lymphoma, presents a formidable diagnostic challenge, particularly in its early stages and in patients with darker skin tones. The initial lesion in this case was diagnosed as cellulitis, a common bacterial skin infection, despite the absence of significant pain, a less typical but documented presentation of cellulitis, which can also manifest as burning, itching, or mere redness and swelling without pronounced tenderness. This case report details the diagnostic journey from initial misdiagnosis to eventual identification of MF in a 57-year-old woman. The report emphasizes the overlapping clinical and histopathological features between MF and more prevalent inflammatory dermatoses, which often lead to diagnostic delays. By including a detailed timeline, comprehensive patient history, explicit diagnostic reasoning, and informed consent declaration, this report aims to highlight the unique barriers to MF diagnosis, especially in individuals with darker skin, and to provide practical insights for clinicians navigating similar diagnostic dilemmas. The rarity and subtlety of early MF, compounded by its ability to mimic benign conditions such as eczema, psoriasis, and cellulitis, underscore the need for heightened clinical suspicion and multidisciplinary collaboration in dermatologic practice.

## Introduction

Mycosis fungoides (MF) is the most common form of cutaneous T-cell lymphoma (CTCL), accounting for approximately 70% of all primary cutaneous lymphomas [1]. It is characterized by the proliferation of malignant T lymphocytes in the skin, typically presenting as patches, plaques, or tumors. The annual incidence of MF is estimated at 3.6 per million persons in the United States, with a male predominance and a median age of onset between 50 and 60 years [1,2]. While the exact etiology remains unclear, genetic predisposition, chronic antigen stimulation, and environmental factors are suspected to play a role in its pathogenesis.

MF has several subtypes, each with distinct clinical and histopathological features. The primary subtypes include the classic Alibert-Bazin MF, folliculotropic MF, pagetoid reticulosis, and granulomatous slack skin [2]. The classic Alibert-Bazin MF is the most common form and is characterized by patches, plaques, and tumors. Folliculotropic MF involves the hair follicles and is often more resistant to treatment compared to other subtypes. It can present with follicular papules, acneiform lesions, and alopecia. Pagetoid reticulosis is a more rare variant that presents as solitary or localized patches or plaques, often on the distal extremities. Finally, granulomatous slack skin is characterized by lax, pendulous skin folds, typically in the axillae or groin [2]. 

MF typically progresses through several clinical stages, each characterized by distinct morphological features. The first stage is the patch stage, described as erythematous, scaly, and often pruritic patches that can mimic eczema, tinea corporis, or other benign dermatoses. Patches are often found on the lower abdomen, upper thighs, buttocks, and breasts. These patches are often subtle and may be present for months or years before a definitive diagnosis is made [1]. The second stage is the plaque stage, described as the development of raised, infiltrated plaques that are usually reddish, purplish, or brownish in color and often with well-defined borders. These plaques may be more intensely erythematous and pruritic than the patches [1]. The third stage is the tumor stage, defined as the appearance of nodular lesions with potential ulceration. Common locations for tumor development include the upper thighs, groin, breasts, armpits, and crook of the elbow. This stage is associated with a poorer prognosis and increased risk of systemic involvement [3]. Finally, the blood can become infiltrated and evolve into Sézary syndrome, characterized by generalized erythroderma, lymphadenopathy, and the presence of malignant T cells (Sézary cells) in the peripheral blood. Blood stages are classified as B0 (absence of significant blood involvement), B1 (low blood tumor burden), and B2 (high blood tumor burden). The presence of these cells can be detected and quantified using flow cytometry and polymerase chain reaction [3,4].

As a rare condition, MF frequently masquerades as benign inflammatory dermatoses, making early diagnosis particularly elusive. The clinical presentation of MF is highly variable, with early lesions often appearing as nonspecific patches or plaques that can be mistaken for more common conditions such as eczema, psoriasis, or cellulitis [2]. This diagnostic ambiguity is further compounded in patients with darker skin tones, where erythema and subtle textural changes may be less apparent, leading to under-recognition and delayed intervention.

The subtle clinical presentation of early-stage MF often leads to misdiagnosis and delayed treatment. Histopathological examination, immunohistochemistry, and molecular studies are crucial for accurate diagnosis. Skin biopsies should be performed on suspicious lesions, and multiple biopsies may be necessary to establish a definitive diagnosis [2]. Immunohistochemical staining can help identify the characteristic T-cell phenotype, including the loss of certain T-cell markers.

This case report details the journey of a 57-year-old female patient diagnosed with early-stage MF, emphasizing the diagnostic challenges, treatment approach, and long-term management of this rare condition. This case highlights the importance of considering MF in the differential diagnosis of persistent, treatment-resistant skin lesions and the role of topical corticosteroids in the management of early-stage disease. Furthermore, it underscores the significance of a multidisciplinary approach to patient care, involving primary care physicians, dermatologists, and potentially lymphoma specialists.

## Case presentation

A 57-year-old woman with a medical history significant for hypertension, type 2 diabetes mellitus, anxiety/depression, and obesity presented for an annual physical examination. Her primary complaint was a worsening, raised, irritative, and scratchy rash on her left shoulder, present for several weeks and unresponsive to over-the-counter steroid cream. She also noted a newly developed red bump on her left elbow that was occasionally itchy.

Initial examination and treatment

**Figure 1:**
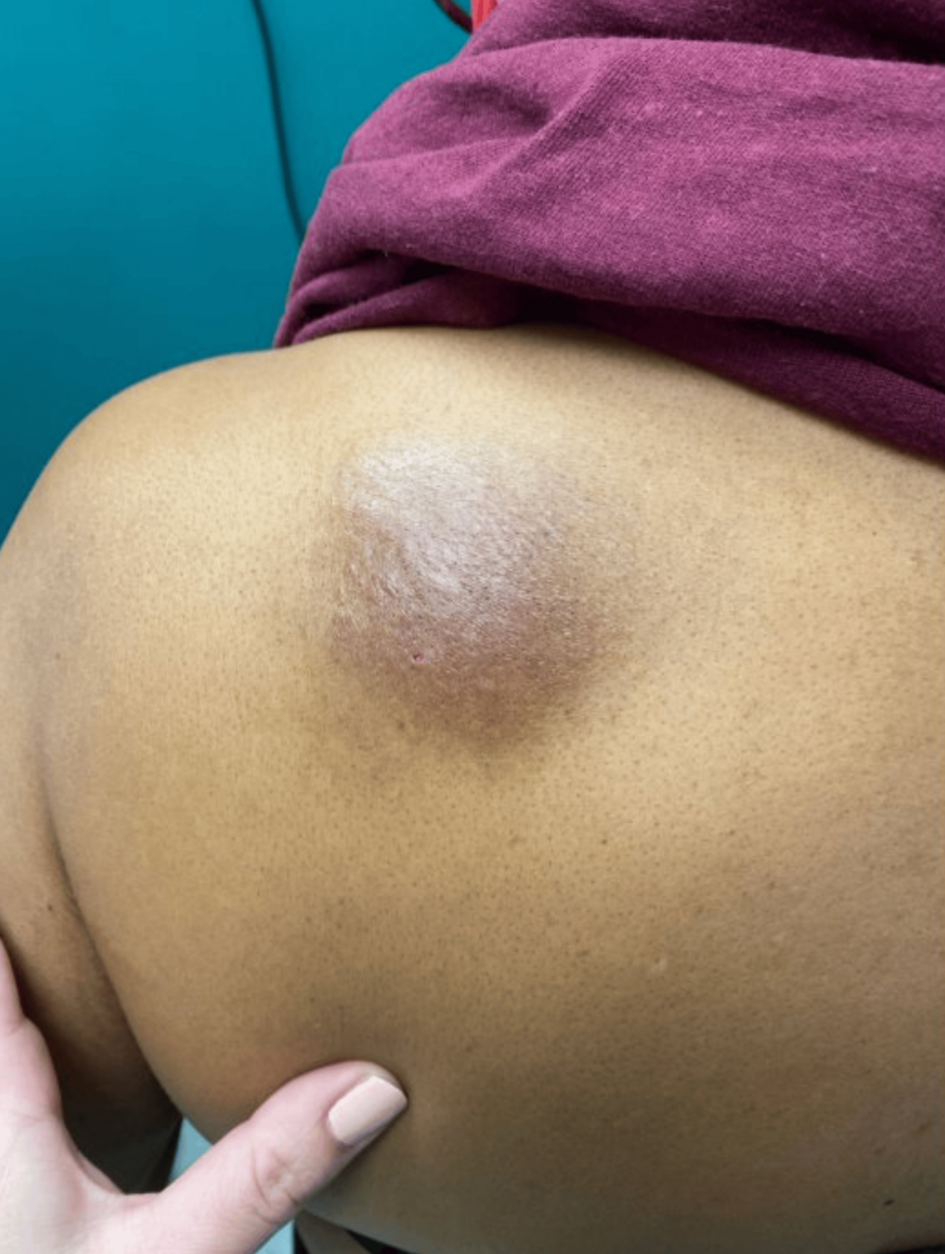
Solitary ovoid rash on the left shoulder. This image depicts the patient presenting with a 4-inch-diameter, poorly demarcated, localized raised hyperpigmented plaque on her left upper back.

**Figure 2:**
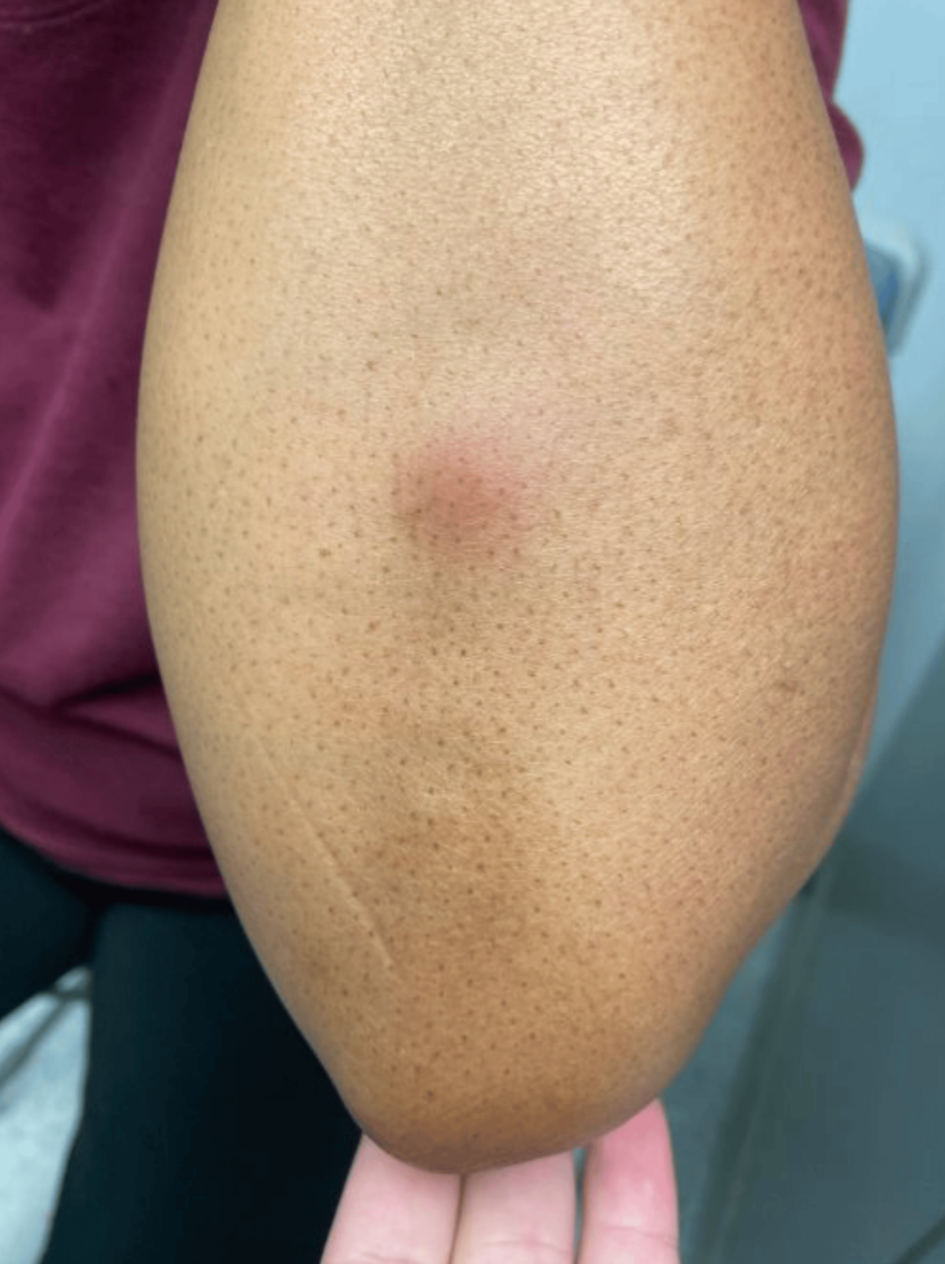
Erythematous papule on the left elbow. This image depicts the patient presenting with a 1-cm-diameter, well-demarcated, localized mildly erythematous papule on her left posterior forearm.

Physical examination revealed a 4-inch, poorly demarcated, raised hyperpigmented plaque on her left upper back (Figure 1) and a 1-cm, well-demarcated, mildly erythematous papule on her left posterior forearm (Figure 2). Based on these findings, a diagnosis of cellulitis of the back (excluding the buttocks) was made, and the patient was prescribed cephalexin 500 mg orally four times daily for seven days. While cellulitis is typically associated with pain, it is important to note that not all cases are painful; some may present with burning, itching, or only visible redness and swelling, as was observed in this patient [5,6]. The absence of classic pain symptoms contributed to diagnostic uncertainty and prompted the consideration of a broader differential diagnosis. The elbow rash was diagnosed as papular urticaria secondary to an insect bite, with a recommendation to take diphenhydramine for symptom control.

Subsequent evaluations and diagnosis

**Figure 3:**
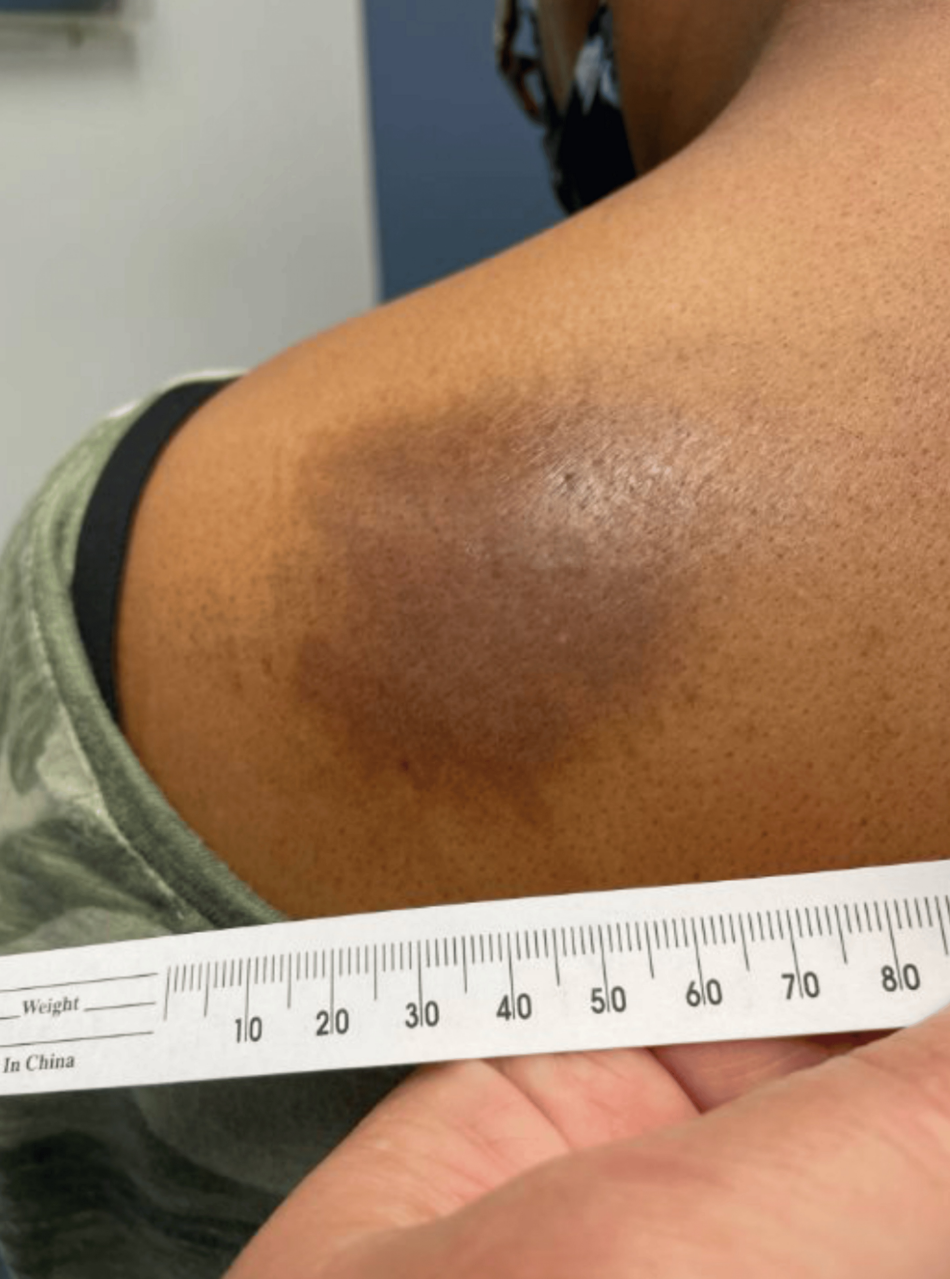
Annular hyperpigmented lesion with peripheral scale on the left upper back.

**Figure 4:**
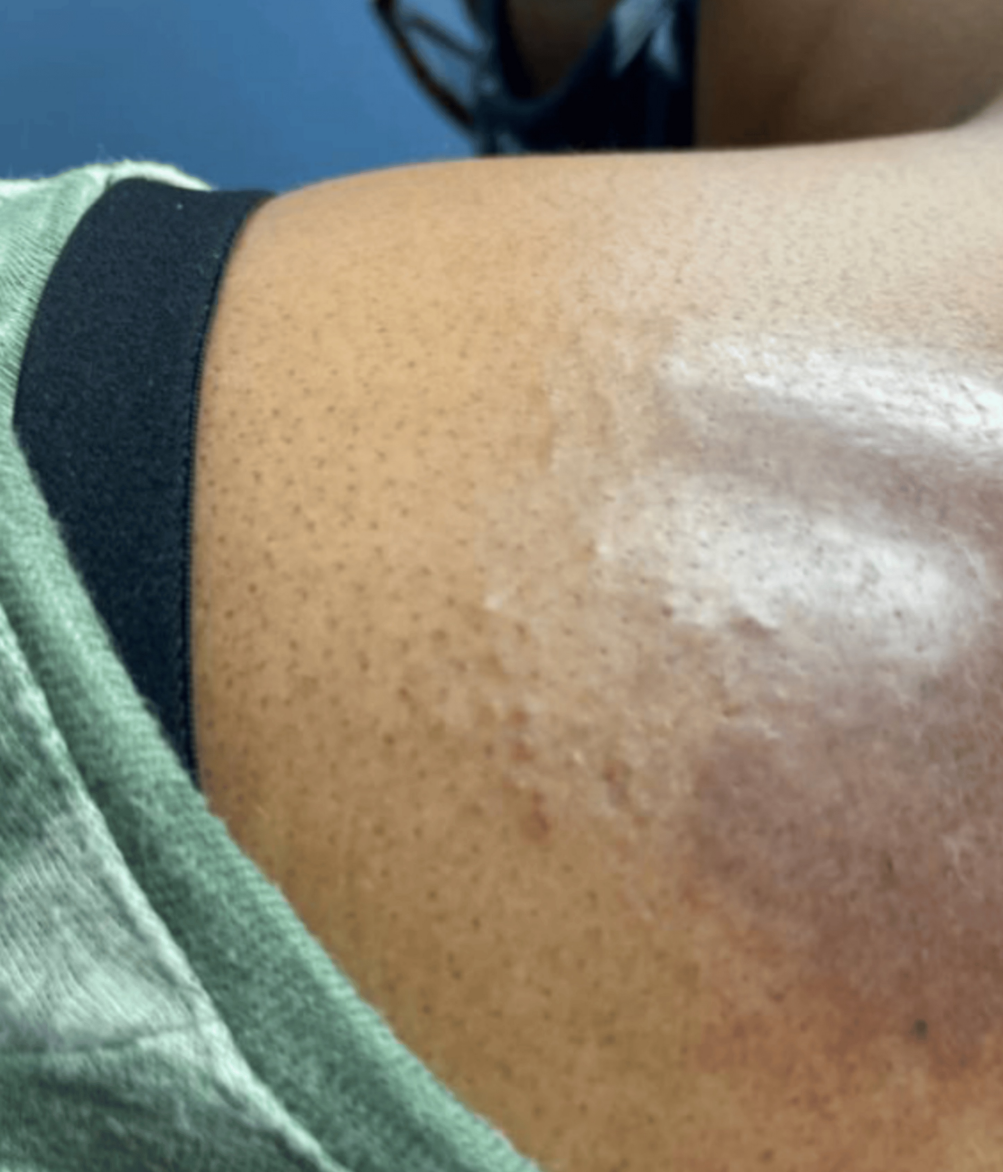
Small papular satellite lesions at the periphery of lesion on the left upper back.

At a follow-up appointment seven weeks later, the patient's elbow papule had resolved, but the upper back plaque persisted and remained itchy despite antibiotic treatment. Examination revealed an annular hyperpigmented lesion with peripheral scale and small papular satellite lesions at the periphery (Figure 3 and Figure 4). The diagnosis was revised to tinea corporis, and clotrimazole 1% cream was prescribed.

After an additional month, the rash was re-evaluated during a hypertension follow-up. The patient reported mild improvement in color and size with the antifungal cream, but the lesion had not fully resolved. Topical antifungals were continued with instructions to call if the rash worsened or changed in character.

**Figure 5:**
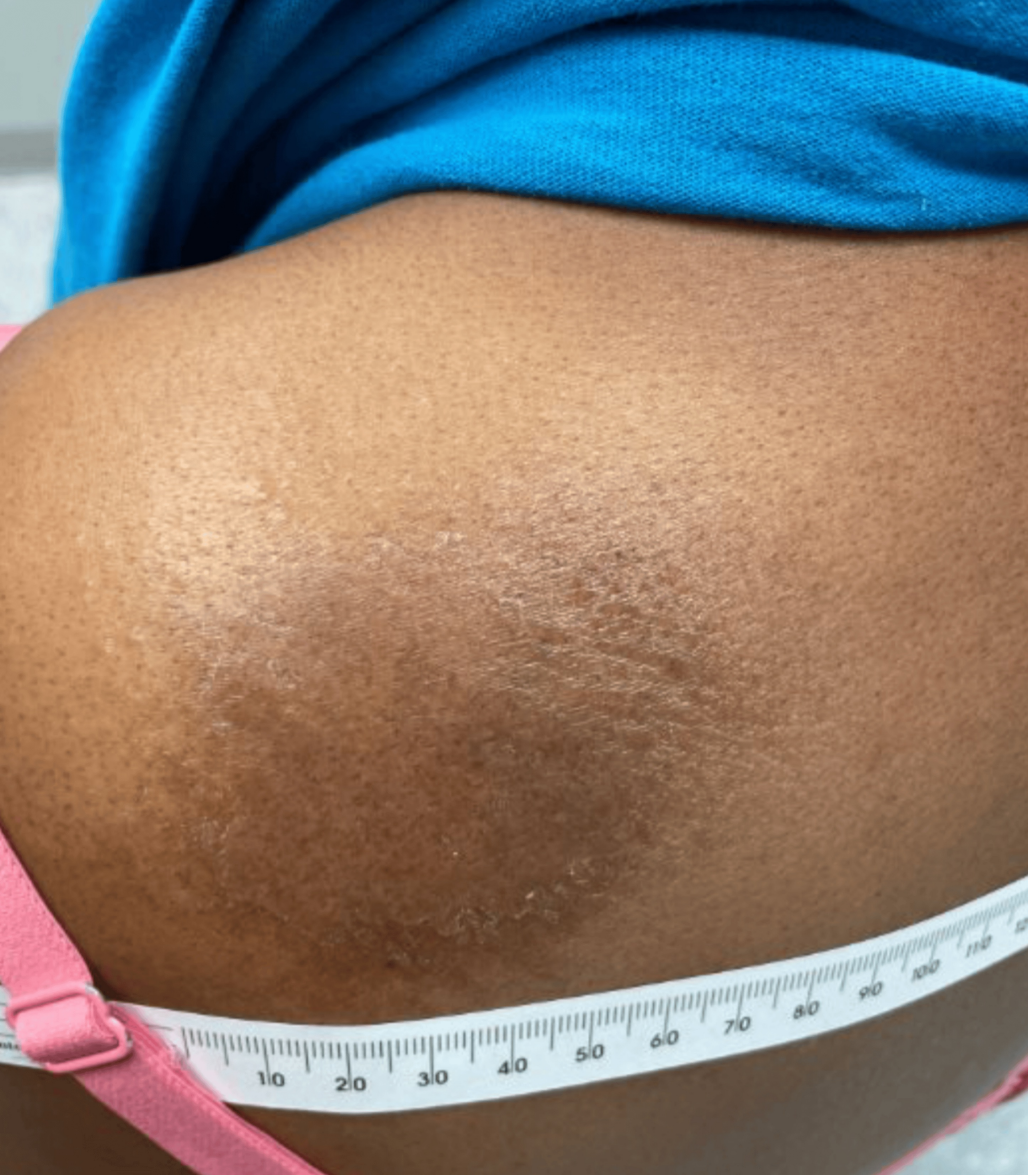
The left upper back lesion improved since the last visit but still has slight scaling and leading edge with central clearing.

Three months later, with persistent symptoms despite topical antifungal treatment, the lesion was re-evaluated again. Physical exam revealed slight scaling and a leading edge with central clearing (Figure 5). An oral antifungal course of fluconazole 150 mg tablet was prescribed, with a recommendation for follow-up if the condition remained unresolved.

Two months later, with no resolution, suspicion for CTCL/MF arose, and a skin punch biopsy was recommended and performed. The sample was sent to pathology, and the patient was referred to dermatology for further evaluation and treatment.

Pathology and final diagnosis

The biopsy sample was immunostained for PanMel, cluster of differentiation 2 (CD2), CD3, CD4, CD5, CD7, CD8, CD10, CD14, CD15, CD19, CD20, CD25, CD30, CD56, CD57, CD68, CD163, granzyme B, Ki-67, lysozyme, myeloperoxidase (MPO), perforin, and T-cell intracellular antigen (TIA). Microscopic examination revealed prominent superficial dermal infiltrates of T cells that seem to express CD2, CD3, and CD4 with loss of expression of CD7. The pathology report concluded an atypical T-cell infiltrate consistent with MF. 

Treatment and outcome

**Figure 6:**
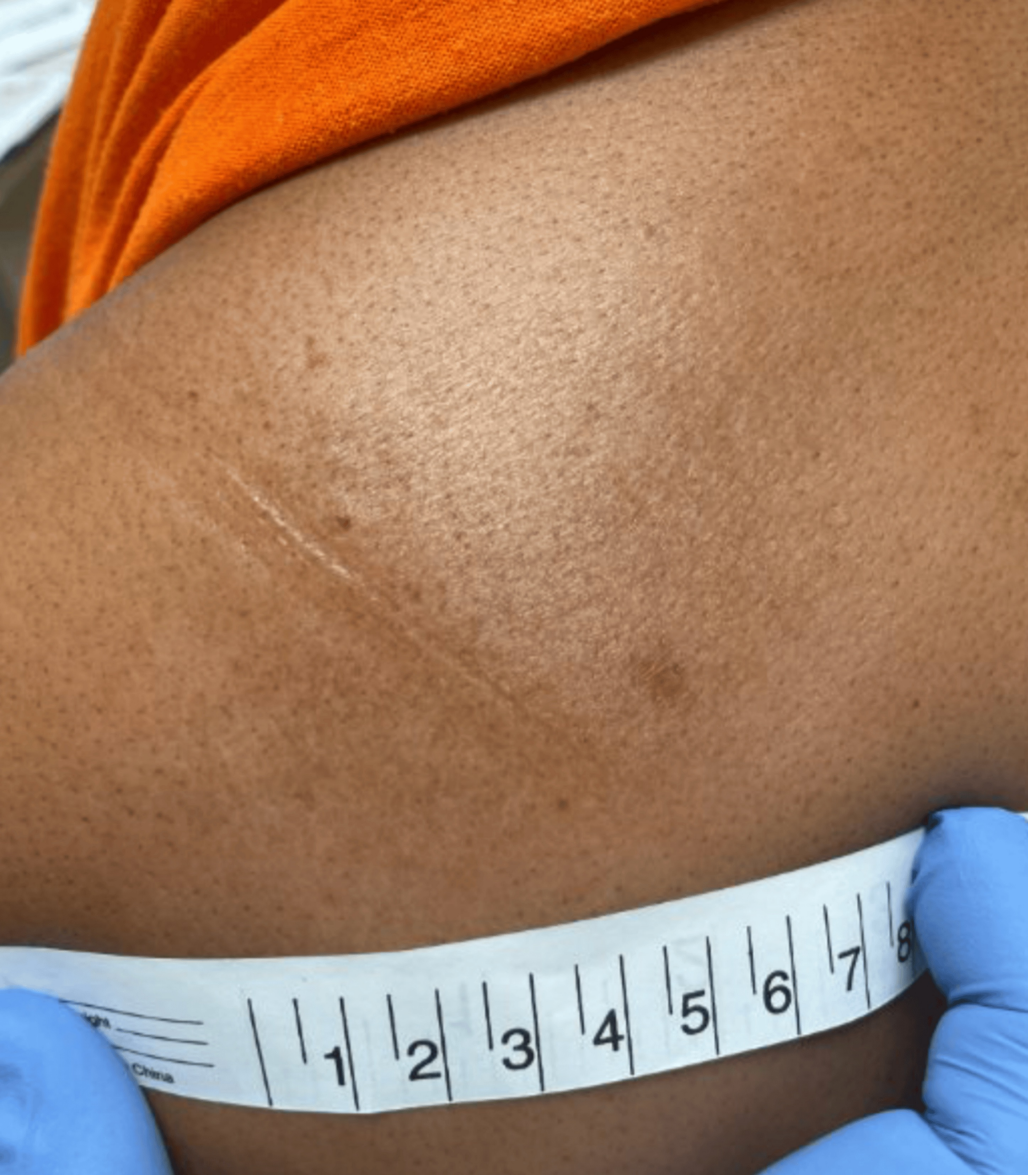
Clinical improvement of mycosis fungoides patch.

Following the diagnosis of MF, the patient was prescribed clobetasol 0.05% ointment by a dermatologist, with a three-month follow-up. At the follow-up, the patient reported using the ointment once per day on weekdays, with perceived improvement and no side effects. Examination revealed a faded MF patch with some residual hyperpigmentation (Figure 6). Continued use of clobetasol was advised. Subsequent follow-up visits at three, six, and 12 months revealed complete clearance of the MF lesion, with some steroid-induced hypopigmentation. Clobetasol was discontinued, and the patient was instructed to return if new or changing lesions appeared.

## Discussion

The initial misdiagnosis of the patient's condition as cellulitis and tinea corporis highlights the difficulty in differentiating early-stage MF from other benign skin conditions. The reduced visibility of erythema and subtle textural changes in darker skin can further delay recognition and referral. This case emphasizes the need for clinicians to maintain a high index of suspicion for MF in patients with persistent, treatment-resistant skin lesions, especially when typical features of common dermatoses are absent or modified by skin pigmentation.

Diagnostic challenges in dark skin

Diagnosing MF in patients with darker skin is particularly challenging. Erythema, a key diagnostic feature in lighter skin, is often less apparent in darker skin tones, leading to delays in recognition and referral. Instead of the readily visible redness, MF may present as subtle changes in pigmentation, texture, or subtle variations in skin tone, which are easily overlooked or misattributed to other conditions. The initial misdiagnoses of cellulitis and tinea corporis in this case highlight this challenge. Cellulitis, a common bacterial skin infection, typically presents with erythema, warmth, swelling, and pain. Tinea corporis, a fungal infection of the skin, often presents with annular, scaly lesions. Both can mimic MF, but the subtle presentation in darker skin further complicates the diagnostic process. The partial response to antifungal treatment further complicated the diagnostic process. Common dermatologic diagnoses, including MF, are frequently missed or misclassified in darker-skinned individuals, underscoring the need for increased awareness and tailored diagnostic strategies in diverse populations. This underscores the importance of maintaining a high index of suspicion for MF in cases of persistent, treatment-resistant skin lesions. In patients who do not respond to standard treatments for common dermatological conditions, a skin biopsy should be considered to rule out MF.

Importance of biopsy and immunohistochemistry

The definitive diagnosis of MF was only achieved through skin biopsy and subsequent immunohistochemical analysis. The characteristic loss of CD7 expression in the atypical T-cell infiltrate was a key finding in confirming the diagnosis [7-9]. In MF, malignant T cells often lose the expression of certain T-cell markers, such as CD7, while retaining the expression of others, such as CD2, CD3, and CD4 [7]. This abnormal immunophenotype can help differentiate MF from benign inflammatory skin conditions. This emphasizes the crucial role of histopathological and immunohistochemical studies in diagnosing early-stage MF.

Effectiveness of topical corticosteroids

The successful management of the patient's MF lesion with clobetasol 0.05% ointment aligns with current treatment guidelines for early-stage, limited MF [10]. Topical corticosteroids are a mainstay of treatment for early-stage MF, as they can effectively suppress the inflammatory response and reduce the proliferation of malignant T cells in the skin. Clobetasol is a high-potency topical corticosteroid that is often used for the treatment of localized MF lesions. The gradual improvement and eventual clearance of the lesion over several months demonstrate the efficacy of potent topical corticosteroids in managing localized MF. While topical corticosteroids are generally well-tolerated, long-term use can lead to side effects such as skin atrophy, telangiectasia, and hypopigmentation [11,12]. Therefore, it is important to use topical corticosteroids judiciously and to monitor patients for potential side effects.

Further research and clinical implications

This case underscores the need for further research into the optimal diagnostic and management strategies for MF, particularly in patients with darker skin. Studies are needed to evaluate the effectiveness of different diagnostic approaches, including non-invasive techniques such as reflectance confocal microscopy and optical coherence tomography, in detecting early-stage MF in diverse skin types. Additionally, research should focus on identifying biomarkers that can aid in the early diagnosis and risk stratification of MF. The clinical implications of this case highlight the importance of incorporating cultural sensitivity and awareness of skin-of-color variations into dermatological training and practice. Clinicians should be educated on the subtle clinical presentations of MF in darker skin and the limitations of relying solely on erythema as a diagnostic marker.

## Conclusions

This case report emphasizes the diagnostic challenges and management considerations for MF, particularly in patients with darker skin tones. Early diagnosis requires a high index of suspicion, careful clinical examination, and the judicious use of skin biopsy with immunohistochemical analysis. Clinicians must be aware of the subtle clinical presentations of MF in diverse skin types and the limitations of relying solely on erythema as a diagnostic marker. Long-term management involves continuous monitoring for disease progression and recurrence, along with patient education and self-examination. Further research is needed to optimize diagnostic and management strategies for MF, particularly in underrepresented populations.
